# Exploring microRNAs, One Cell at a Time

**DOI:** 10.3390/ncrna11060073

**Published:** 2025-10-22

**Authors:** Jessica Kreutz, Tijana Mitić, Andrea Caporali

**Affiliations:** Centre for Cardiovascular Science, The Institute for Neuroscience and Cardiovascular Research, The University of Edinburgh, 47 Little France Crescent, Edinburgh EH16 4TJ, UK; j.kreutz@sms.ed.ac.uk (J.K.); tijana.mitic@ed.ac.uk (T.M.)

**Keywords:** microRNA, single-cell sequencing, bioinformatics, spatial transcriptomics

## Abstract

The emergence of single-cell sequencing and computational analysis has dramatically improved our understanding of cellular diversity and gene expression dynamics. The rapid advancement of high-throughput omics technologies has led to an exponential growth in biological data. However, many gene regulatory processes at the single-cell level remain underexplored, especially those regulated by post-transcriptional mechanisms involving microRNAs (miRNAs). miRNAs are essential regulators of gene expression, affecting cellular functions in both normal and disease states. Recent innovations, such as single-cell gene expression profiling and bioinformatic analysis, have enabled comprehensive studies that uncover previously hidden miRNA profiles. In this context, we present experimental tools and computational methods for analysing cell-specific miRNA abundance and investigating their mechanisms. These approaches are expected to reveal the complex nature of miRNA biology and, more broadly, enhance our understanding of life sciences and diseases.

## 1. Introduction

MicroRNAs (miRNAs) are small non-coding RNA molecules, approximately 22 nucleotides in length. They function as essential regulators of gene expression after transcription. miRNAs play crucial roles in various biological processes, including diseases, stress responses, and development, and are currently being investigated for their potential therapeutic applications [1].

The quantification of miRNAs in experiments primarily relies on bulk small RNA sequencing techniques. However, this approach fails to account for the role of miRNAs in cell heterogeneity. It does not consider how the landscape of miRNA expression influences regulation in rare cell populations within diseases. Although recent developments have made it possible to perform more accurate and efficient sequencing and mapping [2], which is essential for determining the complexities of miRNA expression, there is a necessity to perform single-cell studies, as they enable researchers to explore cellular behaviour and interactions in a more detailed manner.

Currently, there is a lack of single-cell miRNA sequencing datasets. Detecting miRNA in single-cell RNA sequencing poses various technical challenges, making the selective enrichment and subsequent sequencing of miRNA molecules a complex task. Challenges associated with adapters, limited input material, and different technical biases during the reverse transcription and amplification processes impede the accurate detection of miRNA in single-cell RNA sequencing [3]. In recent years, several approaches have been investigated, including the removal of excess adapters [4], the use of degenerate bases in adapters [5] and polyadenylation [6].

**Figure 1:**
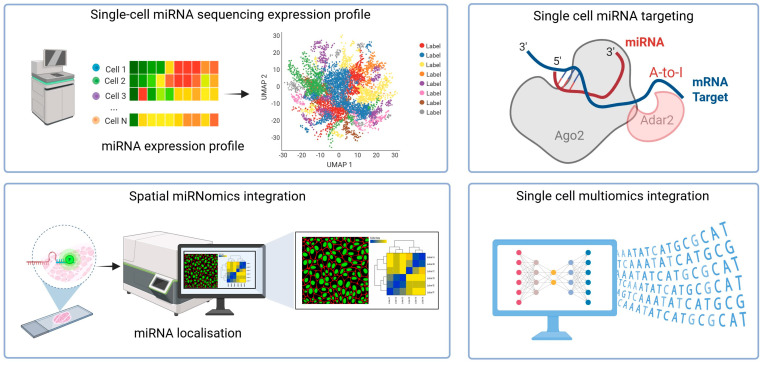
Outline of the experimental workflow for characterising miRNA at the single-cell level.

In this perspective article, we discuss the latest advancements in miRNA sequencing technology and single-cell analysis, which represent a significant step forward in our understanding of the complexities of miRNA regulation and expression in various biological contexts. The experimental setup for these advancements is illustrated in Figure 1.

## 2. Single-Cell microRNA Sequencing

Single-cell miRNA sequencing is an emerging approach that quantifies miRNA expression in individual cells, thereby uncovering heterogeneity that is often masked in bulk analyses.

Recent studies by Hücker et al. [7], Wang et al. [8] and Li et al. [9] have highlighted this recent advance, providing new quantitative and qualitative grounds for single-cell sequencing strategies that facilitate sensitive and high-resolution measurement of miRNA. The advantages and limitations of the novel approaches are summarised in Table 1.

Hücker et al. [7] conducted the first comprehensive comparison of single-cell small RNA sequencing protocols, evaluating nineteen methods to determine the most effective for miRNA sequencing, including ligation-based protocols later referred to as Sandberg protocol I (SB) [4], Sandberg protocol II (SBN) [12] and Sandberg protocol II CleanTag(SBN_CL) [13]. The SB protocol utilised adapters for all RNA species that possess 5′ phosphate and 3′ hydroxyl groups, regardless of their size [4]. Additionally, oligonucleotides were designed to remove the highly abundant 5.8S rRNA. An enzymatic digestion step was also included to minimise the formation of adaptor dimers. Unique molecular identifiers were attached to the 5′ adapters to address PCR stochasticity and facilitate the quantification of RNA molecules. Importantly, no RNA size selection step was conducted during the experiment, and small RNAs were identified through computational analysis. The same authors published an optimised version of the protocol (SBN) in which enzymes and amplification conditions were modified [12]. Finally, in the SBN_CL protocol, the 3′ and 5′ CleanTag adapters from Shore et al. [13] were used in the SBN protocol. Overall, Hucker et al. demonstrated that both the SB and SBN_CL protocols performed exceptionally well in key metrics, including read mapping and miRNA detection rates. Additionally, they demonstrated that advancements in single-cell sequencing are likely to broaden the current understanding of miRNAs and enable discoveries with significant implications for both research and clinical applications.

Wang et al. [8] introduced a half-cell genomics method that splits a single cell lysate, enabling the simultaneous co-sequencing of miRNAs and mRNAs. This strategy overcomes previous challenges in jointly profiling both RNA types at single-cell resolution, achieving high reproducibility and success rates. In this protocol, a single cell was lysed, and the lysate was split evenly into two half-cell fractions, with each fraction subjected to either miRNA or mRNA transcriptome sequencing. Single-cell lysate was heated to release miRNAs for sequential ligation reactions with 3′- and 5′-molecular adaptors. The ligated products were then reverse transcribed and amplified for library preparation and next-generation sequencing. By analysing these paired half-cell profiles, the authors observed significant inverse correlations between highly expressed miRNAs and their predicted mRNA targets, supporting a post-transcriptional regulatory mechanism. This relationship suggests that heterogeneity in miRNA expression can shape individual cell transcriptomes, contributing to non-genetic variability among phenotypically identical cells. Beyond identifying miRNA–target interactions, the method also enables the inference of upstream regulatory pathways that influence miRNA expression. These findings position single-cell miRNA-mRNA co-sequencing as a powerful strategy for dissecting miRNA-mediated post-transcriptional regulation and exploring how dynamic miRNA expression in single cells underlies intercellular heterogeneity.

Recently, Li et al. [9] developed a parallel single-cell small RNA and mRNA co-profiling method called PSCSR-seq V2, which enables the analysis of miRNA and mRNA co-expression in a large number of cells. This method is highly sensitive for miRNA analysis and provides extensive mRNA data. The study identified age-associated miRNAs, including miR-29, as a conserved marker for immunosenescence. PSCSR-seq V2 offers valuable capabilities for functional studies in both clinical and basic biological research.

These studies exemplify how emerging single-cell miRNA sequencing technologies are transforming our ability to resolve miRNA regulation and cellular heterogeneity, offering increasingly high-resolution insights into miRNA profiles associated with specific tissues and disease states, and extending the reach of analyses to rare and clinically meaningful cell populations.

## 3. Spatial microRNA Detection

Unlocking the spatial landscape of miRNAs at single-cell resolution, defined as spatial miRNomics [14], in clinically relevant samples has remained a formidable challenge. Bai et al. [10] addressed this issue by presenting Patho-DBiT. This innovative spatial transcriptomics platform enables highly sensitive and spatially resolved detection of miRNAs in archival formalin-fixed paraffin-embedded tissues. These samples are essential in clinical pathology, but their intrinsic RNA fragmentation has limited their use in transcriptome-wide profiling (see Table 1 for description, advantages and limitations). Patho-DBiT overcomes this barrier by integrating in situ polyadenylation with spatial microfluidic barcoding, enzymatically adding poly(A) tails to the fragmented transcripts. This approach facilitates the comprehensive capture of coding and non-coding RNAs, including precursor and mature miRNAs, which have historically been challenging to profile spatially due to their small size and lack of poly(A) tails. Alongside miRNAs, the platform concurrently profiles gene expression, alternative splicing, RNA editing events such as A-to-I conversion, and single-nucleotide variants, offering a multilayered view of transcriptomic architecture. Coupled with iStar computational refinement, Patho-DBiT achieves single-cell-level spatial transcriptome mapping, resolving fine-grained cellular heterogeneity and tumour microenvironmental organisation. By applying this platform to a mucosa-associated lymphoid biopsy, the authors detected over 1300 miRNAs, revealing distinct expression patterns between tumour and non-tumour regions as well as across cell types. Their spatial profiling also uncovered miRNA regulatory networks linked to key oncogenic pathways implicated in tumorigenesis. Furthermore, Patho-DBiT facilitated spatial mapping of tumour evolution at the cellular level. This analysis demonstrated how B-cell tumour cells engage distinct microenvironments to support proliferation and survival, while capturing the spatial molecular dynamics underlying this progression.

By overcoming technical challenges in detecting miRNA from archival clinical tissues, this work represents a significant step forward in spatially resolving miRNA biology at the single-cell level. It opens new opportunities to unravel miRNA regulatory networks involved in spatial tumorigenesis and lays the foundation for incorporating spatial miRNA profiling into future diagnostic and therapeutic strategies.

## 4. MicroRNA Targeting at the Single-Cell Level

Achieving transcriptome-wide detection of miRNA–target interactions at single-cell resolution has been made possible with agoTRIBE, a novel method introduced by Sekar et al. [11]. This approach fuses Argonaute2 with a hyperactive RNA-editing domain of ADAR2, creating a fusion protein that leverages endogenous miRNAs to locate and bind their mRNA targets in living cells (see Table 1 for pros and cons of this approach). Upon binding, the editing domain catalyses detectable adenosine-to-inosine editing events that can be captured via single-cell RNA sequencing. Traditional CLIP-seq approaches for identifying miRNA targets require millions of cells and complex workflows, rendering them impractical for single-cell applications and potentially concealing significant cell-to-cell differences. In contrast, agoTRIBE enables straightforward, antibody-free transfection down to the individual cell level, reducing experimental costs and duration while increasing editing sensitivity. Significantly, the miRNA targets identified by agoTRIBE are enriched for evolutionarily conserved binding sites and exhibit evidence of functional miRNA repression, supporting the method’s ability to capture biologically meaningful interactions. Applying this method at single-cell resolution, Sekar et al. demonstrated pronounced heterogeneity in miRNA targeting across individual cells, revealing dynamic differences between cells at distinct cell cycle phases and differentiation stages during K562 leukaemia cell maturation into erythroid precursors. As each miRNA’s function is defined by its unique repertoire of targets, the ability of agoTRIBE to record targeting events at single-cell resolution represents a significant advance toward fully understanding the roles of miRNAs. By using RNA editing as a proxy for miRNA–mRNA interactions, this method provides a powerful means to infer functional targeting within individual cells.

This cellular granularity opens the door to disentangling miRNA activity in complex tissues where diverse cell types coexist, making it possible to resolve cell type-specific targeting that would otherwise be masked by tissue-level averages, and to uncover hidden diversity within seemingly homogeneous cell populations. Moreover, the method’s simultaneous output of RNA abundance alongside target detection supports future efforts to link miRNA activity to downstream transcriptomic effects in single cells.

## 5. Bioinformatics Approaches

As single-cell technologies for miRNA detection and targeting advance, new bioinformatics tools are being developed to address the unique complexity of these datasets. Contributions by Engel et al. [15] and Herbst et al. [16], demonstrated computational innovations specifically designed for single-cell miRNA analysis, providing deeper insights into the biology of miRNA (summarised in Table 2).

Recognising the absence of a web server offering a streamlined workflow and intuitive graphical interface for single-cell miRNA-seq data, Engel et al. [15] developed SingmiR to address key accessibility and analysis challenges in this emerging area. This user-friendly platform is specifically designed to process and analyse single-cell miRNA sequencing data, allowing users without computational expertise to perform comprehensive analyses. Unlike existing bulk miRNA and single-cell mRNA tools that do not support the adapter and UMI layouts specific to single-cell miRNA protocols, SingmiR accommodates these parameters and guides users step-by-step through protocol-specific read trimming, alignment, automated quality control, and normalised miRNA quantification. The platform also provides built-in downstream analyses, including dimension reduction (such as PCA and UMAP), hierarchical and correlation-based clustering, and interactive differential expression, with metadata that enable user-defined comparisons. Validated on published single-cell miRNA-seq datasets, SingmiR demonstrates its practical utility. By integrating these capabilities into an intuitive platform, SingmiR lowers technical barriers and broadens access to advanced analytics, empowering researchers to efficiently prototype and benchmark new single-cell miRNA sequencing protocols and extract meaningful biological insights from single-cell miRNA datasets.

Complementing such advances in data processing and quantification, Herbst et al. [16] developed miTEA-HiRes, a Python-based package designed to infer miRNA activity from single-cell and spatial transcriptomics data by analysing the expression of their targets. The method leverages experimentally validated miRNA–target interactions curated from miRTarBase and employs a minimum hypergeometric test to compute miRNA activity scores across single-cell RNA-seq and spatial datasets. Offering both total and comparative activity modes, miTEA-HiRes facilitates the construction of high-resolution miRNA activity maps that reveal miRNA heterogeneity across diverse cell populations. It also enables the detection of miRNAs that are differentially active between cells under varying conditions, thereby capturing activity signals that are often missed in conventional gene expression datasets. Its application to single-cell RNA-seq data from peripheral blood mononuclear and cerebrospinal fluid cells of multiple sclerosis patients and controls, as well as from migratory and static breast cancer cells, highlights its ability to uncover activity patterns that distinguish disease states and cell types. Furthermore, validation using single-cell induction data demonstrates the method’s robustness in capturing miRNA activity at cellular resolution. At the same time, its independence from large pre-training datasets positions it to improve accessibility. By identifying differentially active miRNAs, miTEA-HiRes may help pinpoint candidate miRNAs that warrant further validation. These candidates could serve as prognostic biomarkers in disease and metastasis, thereby expanding the utility of single-cell transcriptomics for biomedical discovery.

Together, these studies demonstrate that bioinformatic approaches are continually evolving to support the expanding field of single-cell miRNA research, providing both higher-resolution insights and increased accessibility for users.

## 6. Conclusions and Future Directions

The body of research conducted in this area underscores the profound impact that single-cell and spatial miRNA sequencing technologies can have on our understanding of molecular pathways that play critical roles in various disease states. By examining miRNA expression at the single-cell level, researchers can identify heterogeneity within cell populations, revealing insights that are often masked in bulk tissue analyses. Spatial sequencing further enhances this approach by preserving the contextual information of where specific miRNAs are expressed within tissue samples, enabling a more comprehensive understanding of cellular interactions and microenvironments.

### 6.1. Multimodal Analysis

Future miRNA research at the single-cell level will depend on integrated approaches that combine co-sequencing and computational analysis to gain a deeper understanding of regulatory mechanisms. The analysis of the extensive and intricate datasets produced from single-cell miRNA sequencing will necessitate the development and utilisation of advanced computational tools. Gondal et al. [17] reviewed the expanding number of bioinformatics approaches adapted for single-cell non-coding RNA (ncRNA) analysis, emphasising how the advent of single-cell RNA sequencing is transforming the computational landscape of the field. The review also highlights the significance of emerging single-cell multi-omics integration tools, such as MOFA+ [18], which combine single-cell RNA-seq data with other molecular data to elucidate the functions of ncRNAs, alongside network-based methods that infer cell-type-specific regulatory networks involving ncRNAs. These bioinformatic strategies can also be exploited to unmask cell-type-specific miRNA signatures.

### 6.2. Clinical Implications of Spatial miRNomics

The spatial biology of miRNAs will be crucial for a comprehensive understanding of their functions and roles in human disease. Spatial miRNomics will not only deepen our knowledge of the underlying mechanisms driving diseases but also pave the way for the development of more targeted and effective therapeutic strategies. As already proposed for spatial transcriptomics [19], spatial miRNomics could lead to significant advancements in precision medicine and biomarker identification, enabling tailored treatments that are more closely aligned with the unique molecular characteristics of individual patients and their specific conditions.

### 6.3. Sequencing Sensitivity and Throughput

Enhancing sensitive and high-throughput methods is essential. This involves improving library preparation techniques and sequencing technologies to accurately detect miRNAs. In this context, the Kirsch lab has developed robust protocols for the enzyme-free preparation of tissue-derived single-cell suspensions [17] and for the preparation of single-cell miRNA sequencing libraries, which can be easily adopted by various laboratories [20]. Additionally, Nanopore sequencing technology offers enhanced sensitivity for identifying low-abundance miRNAs and their isoforms [21]. Finally, bias in small RNA library preparation can significantly impact data accuracy, skewing the representation of miRNAs. Studies show that this bias primarily occurs during the ligation of single-stranded adapters, which is exacerbated by 2′-O-methyl modifications on the 3′ terminal nucleotide. A novel library preparation method using randomised splint ligation with a cleavable adapter effectively addresses these challenges by reducing bias and enhancing the sensitivity of small RNA sequencing across various RNA types, including miRNAs [22].

### 6.4. Evolution of Single-Cell microRNA-mRNA Co-Sequencing Techniques

Studying miRNAs in isolation provides an incomplete understanding of gene regulation. Integrating miRNA sequencing with mRNA sequencing, and potentially other omics data from the same single cell, will offer a more comprehensive perspective on gene regulatory networks. Recently, Velut et al. [23] proposed an improved and rigorous methodology with shared codes to perform such analysis. The study reported the optimal parameters for identifying a statistically significant anticorrelation between a miRNA and its predicted targets and advised on how to analyse such co-sequencing datasets.

### 6.5. Data and Code Sharing

As scRNA-seq technology continues to revolutionise our understanding of complex biological systems, data and code sharing as well as transparency have become increasingly critical to the advancement of the field. The deposit of scRNA-seq data and the sharing of analysis codes are essential for fostering a collaborative, transparent, and innovative research environment. These practices not only enhance the credibility and reproducibility of scientific work but also enable the community to address complex biological questions more effectively. By embracing data sharing, researchers can ensure the progress of scRNA-seq as a transformative tool in the life sciences. The use of online software can facilitate the use of publicly available scRNA-seq datasets, thus advancing our understanding of cellular biology and disease mechanisms. DISCO (Deeply Integrated human Single-Cell Omics) [24] is a newly developed database that offers harmonised human gene expression data and associated metadata for public use. Unlike other databases, DISCO processes data from FASTQ files, available for 95% of its datasets, rather than relying on original read count tables. This involves re-aligning raw reads to a single reference genome and using a consistent gene annotation file, which minimises batch effects and improves data integration. Researchers can access a wealth of public datasets, explore cellular diversity across tissues and diseases, and analyse their own data alongside these resources.

## Figures and Tables

**Table 1:** Summary of single-cell and spatial microRNA sequencing techniques.

Method	Summary	Advantages	Limitations	Ref
**Sandberg** **Protocol I (SB)**	A small RNA sequencing protocol for single cells that captures miRNAs using sequential 3′ and 5′ adapter ligation.	Demonstrated strong re-producibility between replicates in single-cell equivalent experiments. Exhibited broad miRNA detection, identifying a high number of protocol-exclusive miRNAs and capturing several distinct miR-NAs from a single-cell equivalent.	As a ligation-based method, it is susceptible to adapter-dimer formation with low-input samples, reducing usable reads. Demonstrated variable miRNA detection between replicates.	[7]
**Sandberg** **Protocol II CleanTag (SBN_CL)**	An “optimised” version of SB that incorporates chemically modified CleanTag adapters to suppress the formation of adapter dimers.	Demonstrated high reproducibility in single-cell experiments, outperforming the SB protocol. Demonstrated robust performance and versatility by successfully profiling miRNAs at the single-cell level in multiple distinct cell lines. Demonstrated high concordance in miRNA detection. Reduces adapter-dimer formation, increasing the yield of informative reads from low-input samples. Designed to be easily automated.	As a ligation-based method, it remains vulnerable to adapt-er-dimer formation. It requires future optimisation because it was validated only on cell lines.	[7]
**Half-Cell** **Genomics** **Approach**	A co-sequencing method where a single cell’s lysate is split into two equal frac-tions, enabling the simulta-neous profiling of both miRNAs and mRNAs from the same cell.	Enables direct analysis of post-transcriptional regulation by simultaneously co-sequencing the miRNA and mRNA transcrip-tomes from the same single cell. Demonstrates a high success rate and excellent reproducibility for profiling both miRNAs and mRNAs from a single cell’s lysate. The half-cell sampling method is validated to ensure that each half’s lysate accurately represents the entire transcriptome of the single cell.	The manual protocol has limited throughput for profiling large numbers of single cells, a limitation the authors suggest could be improved by integration into microfluidic systems. The method necessitates a complex, multi-step lysis protocol with increased sample handling. The method’s capability to detect miRNA–target interactions depends on high expression levels of both the miRNA and its target, limiting its use for studying low-abundance total RNA.	[8]
**PSCSR-seq V2**	A parallel, barcoded sin-gle-cell coprofiling method that integrates a SMART-seq reaction into the PSCSR small RNA workflow, ena-bling high-sensitivity se-quencing of miRNAs along-side rich mRNA information from thousands of individ-ual cells.	Highly scalable, allowing high-throughput co-profiling in large numbers of single cells. Offers highly sensitive single-cell miRNA detection. Overcomes the difficulty of an-notating cells in heterogeneous samples (e.g., tissues). Enables finer-resolved classifications of cells than is possible using either data type alone by integrating miRNA and mRNA pro-files, achieving a more precise separation of cell subpopulations.	Lower miRNA detection sensitivity than methods dedicated solely to small RNA profiling. Detects fewer mRNA species per cell compared to other half-cell genomics approaches.	[9]
**Patho-DBiT**	A spatial transcriptomics platform that conducts whole transcriptome se-quencing on archival for-malin-fixed paraf-fin-embedded tissues using in situ polyadenylation for diverse RNA capture and microfluidic barcoding for spatial mapping.	Enables high-sensitivity spatial transcriptomics in archived for-malin-fixed paraffin-embedded tissues by overcoming RNA fragmentation, proven effective on specimens stored for over five years. Provide functional analysis by spatially co-profiling both pre-cursor and mature microRNA strands together with the complete mRNA transcriptome, thereby enabling the mapping of microRNA regulatory networks.	Patho-DBiT alone lacks actual single-cell resolution, requiring computational refinement. The technology faces low coverage at the pixel level for some analyses, a limitation the authors explicitly mention regarding alternative splicing.	[10]
**agoTRIBE**	A method for detecting miRNA–target interactions in single cells involves fusing Argonaute2 with a hyper-active ADAR2 RNA-editing domain. This fusion allows endogenous miRNAs to edit target mRNAs, producing A-to-I (read as A-to-G) marks that can be identified through single-cell RNA sequencing.	Low cell input requirements to the single-cell level by eliminating the costly and inefficient anti-body-based isolation. Simplified and faster workflow that avoids both laborious protocol steps. Captures interactions directly in living cells, preserving the native context and avoiding artefacts introduced by cell lysis required for immunoprecipitation-based methods. Generates multi-modal data by simultaneously capturing miRNA and target interactions in the same single cell.	Fails to resolve exact miRNA binding positions, limiting its utility to the identification of target transcripts rather than specific interaction sites.	[11]

**Table 2:** Summary of bioinformatics approaches.

Method	Summary	Advantages	Limitations	Ref
**SingmiR**	A user-friendly web server with an intuitive interface provides a comprehensive bioinformatics pipeline for single-cell miRNA-seq data, encompassing everything from raw read pre-processing and miRNA quantification to di-mension reduction and dif-ferential expression analysis.	Freely accessible web server with a self-explanatory interface and no login requirement. Integrates the entire bioinformatics workflow on a single platform, automating the pipeline from rapid (pre)processing and quanti-fication. Facilitates the rapid prototyping and benchmarking of new sin-gle-cell miRNA-seq protocols.	Currently supports only low-throughput sequencing data, defined as up to a few hundred thousand reads per cell.	[15]
**miTEA-HiRes**	A statistical method inferring miRNA activity from sin-gle-cell and spatial tran-scriptomics data based on validated target expression patterns. It enables the creation of spatial activity maps and the assessment of overall activity, as well as differential analysis in single-cell data.	Provides robust quality of results without relying on large pre-training datasets. Offers a flexible, unified approach to infer miRNA activity from both single-cell and spatial tran-scriptomics datasets, a capability that previous tools do not address. Offers ease of use through its im-plementation as a user-friendly, pip-installable Python package that provides an end-to-end anal-ysis pipeline.	As an inference method, its findings do not serve as direct confirmation of miRNA roles. The software is computation-ally intensive, leading the au-thors to recommend running miTEA-HiRes on a multicore machine. Less suitable for comparing very small cell subpopulations. The tool’s analysis is limited by its reliance on a precomputed set of miRNA-target interac-tions, curated from the miRTarBase database.	[16]

## Data Availability

No new data were created.

## Abbreviations

The following abbreviations are used in this manuscript: microRNA (miRNA), non-coding RNA (ncRNA).
